# The effect of acute pre-workout supplementation on power and strength performance

**DOI:** 10.1186/s12970-016-0138-7

**Published:** 2016-07-16

**Authors:** Nic Martinez, Bill Campbell, Madison Franek, Laura Buchanan, Ryan Colquhoun

**Affiliations:** Exercise Science Program, Performance & Physique Enhancement Laboratory, College of Education, University of South Florida, Tampa, FL 33620 USA

**Keywords:** Sports nutrition, Dietary supplement, Pre-workout, Anaerobic power, Human performance

## Abstract

**Background:**

Consumption of pre-workout dietary supplements by both recreational and competitive athletes has increased dramatically in recent years. The purpose of this study was to determine the acute effects of a caffeine-containing pre-workout dietary supplement on various measures of performance including anaerobic power, upper and lower body power, and upper body strength in recreationally trained males.

**Methods:**

Thirteen males (mean ± SD age = 24 ± 6 yrs; height = 180.3 ± 5 cm; body mass = 83.4 ± 9 kg) participated in this investigation in which they reported to the laboratory on four separate occasions, each separated by one week. Each subject underwent an initial familiarization session on week one followed by baseline (BA) performance testing on week two. Performance testing included a medicine ball put (MBP) to determine upper body explosive power, vertical jump test (VJ) to determine lower body explosive power, one-rep maximum bench press (1-RM) for determining upper body strength, and a Wingate Anaerobic Power Test (WAnT) to determine measures of anaerobic power. On week three, subjects were randomly assigned to ingest either a pre-workout supplement (SUP) or a placebo (PL) and again complete the performance testing protocol. Subjects were provided with the crossover treatment on the fourth and final week. Performance testing commenced 20-minute following ingestion of both treatments, which was similar to previous investigations.

**Results:**

Significant differences in anaerobic peak power relative to the WAnT were observed following ingestion of the SUP (782 ± 191 W) in comparison to the PL (722 ± 208 W; *p* = 0.003; effect size = 0.30) and BA (723 ± 205 W; *p* = 0.011; effect size = 0.28). Significant differences were also observed for anaerobic mean power following ingestion of the SUP (569 ± 133 W) in comparison to the PL (535 ± 149 W; *p* = 0.006; effect size = 0.24) and BA (538 ± 148 W; *p* = 0.020; effect size = 0.22). No significant differences between trials were observed for upper body power, lower body power, or upper body strength.

**Conclusions:**

Ingestion of the pre-workout dietary supplement led to significant improvements in anaerobic peak and mean power values in comparison to the placebo and baseline treatments. No improvements were observed in upper and lower body power or upper body strength. Taken prior to exercise, a caffeine-containing pre-workout dietary supplement may improve anaerobic power performance.

## Background

Pre-workout supplementation has continued to rise in popularity among both recreational and athletic populations interested in improving performance. Approximately 70 % of young adults consume at least one nutritional supplement, and one of the most popular categories of nutritional supplements consumed is pre-workout energy drinks [1, 2]. Specifically, 30 % of young adults consume these energy-boosting supplements on a regular basis; this rate of consumption places pre-workout supplements second in usage only behind that of multivitamins [3]. It has been reported that legal pre-workout supplements appeal to physically active individuals seeking to improve performance as an alternative to illegal performance enhancing drugs [4]. However, many of the claims made by manufacturers and consumers of pre-workout supplements have not been fully validated within the context of performance testing. Therefore, further investigation of these ergogenic aids and their potential impact on various measures of performance is of importance to both the scientific and athletic communities.

Most pre-workout supplements contain a proprietary blend of ingredients that claim to produce performance benefits when ingested simultaneously. Caffeine, a mild nervous system stimulant and principal active ingredient in most pre-workout supplements, has been shown to enhance performance for endurance sports by increasing time to exhaustion, preserving muscle glycogen content, delaying perceptions of fatigue, and decreasing perceptions of pain and effort [4–9]. While caffeine functions through a variety of mechanisms, its ability to inhibit action at the adenosine receptor site is believed to be the primary factor responsible for decreasing the perception of pain and effort, and the resulting improvement in endurance performance [4, 10]. Fatigue during exercise appears to be delayed by caffeine’s ability to influence exercise metabolism through the improvement of fat oxidation, which in turn facilitates the preservation of muscle glycogen content [11].

Caffeine has also been shown to produce a favorable increase in anaerobic peak power measured during a WAnT [12]. It has been suggested that caffeine may augment strength and power performance through greater motor unit firing rates, increased calcium release from the sarcoplasmic reticulum, and surges in nitric oxide concentrations, collectively working to produce stronger muscle contractions [13, 14]. While some of the scientific literature has reported that caffeine is able to improve anaerobic power performance, there are also reports that suggest it is ineffective for power enhancement [15, 16]. In a comprehensive review on this topic, Goldstein and colleagues stated “the literature is equivocal when considering the effects of caffeine supplementation on strength-power performance, and additional research in this area is warranted” [17].

Manufacturers of pre-workout supplements often combine caffeine with other select ingredients in an attempt to produce a synergistic effect with ergogenic potential. The combination of multiple ingredients such as caffeine, creatine, amino acids, taurine, and glucuronolactone has been shown to delay fatigue and improve the overall quality of resistance training sessions [18]. Unfortunately, in many commercially available sports nutrition dietary supplements, the ingredient dosages are understudied in terms of their combinations and under-dosed in terms of their quantity.

Recently, a caffeine-containing pre-workout supplement (Assault™, MusclePharm, Denver, CO, USA) has been marketed as an ergogenic aid for improving performance through increased energy, focus and power. This pre-workout dietary supplement contains a variety of select ingredients such as caffeine, branched chain amino acids (BCAAs), creatine, beta-alanine, citruline malate, arginine, vitamin B-6, vitamin B-12, and other ingredients. Previous investigation of this pre-workout dietary supplement revealed multiple performance enhancing benefits including improved lower body strength, choice reaction time, focus and alertness [14]. However, no studies to date have examined the effect of this pre-workout supplement on upper body strength and power performance. The purpose of this investigation was to compare the effects of a commercially available pre-workout supplement and a placebo treatment on various measures of performance including the primary outcome of anaerobic power and secondary outcomes of upper and lower body explosive power, and upper body strength.

## Methods

### Subjects

Thirteen males (mean ± SD age = 24 ± 6 yrs; height = 180.3 ± 5 cm; body mass = 83.4 ± 9 kg) volunteered to participate in the study. The sample size is a reflection of related research [12], and is based on an anticipated medium-to-large effect size (i.e., ES = 0.5 to 0.8), a power level of 0.8, and an alpha criterion of 0.05 for measures of anaerobic peak power, this study’s primary dependent variable. All procedures relative to this study were approved by the University of South Florida’s Institutional Review Board for the protection of human subjects. All subjects completed a health history questionnaire and signed a written informed consent prior to any supplementation or testing. Subjects were recreationally trained, low-risk, and met the minimum recommended standards of physical activity set forth by the American College of Sports Medicine [19]. Subjects reported engaging in one or more of the following recreational sport activities: running, cycling, tennis, swimming, and resistance training. Subjects were required to avoid consumption of any pre-workout supplements for at least 2 weeks prior to beginning the study as well as throughout the duration of the investigation, unless provided by laboratory study staff as part of the intervention.

### Study design

This study utilized a randomized, double-blinded, crossover design. Subjects reported to the laboratory on four separate occasions with each visit separated by one week. All subjects were instructed to refrain from consuming food, caloric beverage or caffeine at least 3 h prior to each trial. Subjects were asked to continue their current training programs throughout the duration of the study. However, subjects were instructed to avoid performing any type of strenuous physical activity for 24 h prior to each trial. All subjects underwent an initial familiarization trial, which included instructions on how to complete a 24 h food log, standardized dynamic warm-up, and completion of the performance testing protocol structured in the following order: MBP, VJ, 1-RM, and WAnT. Subjects underwent a baseline trial during their second visit to the laboratory in which they submitted a completed 24-hour food log, which was used to replicate caloric and macronutrient intake for the 24 h prior to future trials. Subjects then completed baseline assessments for height, weight, and blood pressure and were guided through a standardized dynamic warm-up followed by completion of the aforementioned performance testing protocol.

**Fig. 1:**
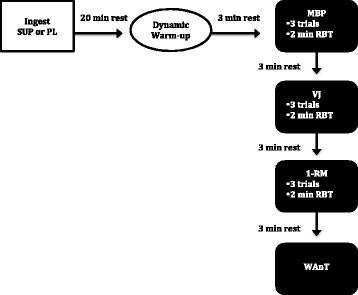
Study protocol. SUP supplement, PL placebo, MBP medicine ball put, VJ vertical jump, 1-RM bench press, WAnT Wingate Anaerobic Power Test, RBT rest between trials

On the third visit, subjects were randomly provided with either the SUP or the PL. On the fourth visit, subjects were provided with the opposite treatment. During both the third and fourth visits to the laboratory, subjects were instructed to sit in a rested state for 20 min following ingestion of either the SUP or PL. The timing of the ingestion of the pre-workout dietary supplement mimicked the protocol utilized by Spradley and colleagues [14]. Following the rest period, subjects were directed to a 5-minute dynamic warm-up station in preparation for physical activity. Subjects were then assessed on upper and lower body power while performing a MBP and VJ, respectively. Following the upper and lower body power assessments, subjects performed a 1-RM strength assessment, which was followed by an anaerobic power test utilizing the WAnT. Each performance assessment was separated by a 3-minute rest period. Two study staff members were present during testing to ensure proper safety, documentation of the assessment, and execution of the protocol. Figure 1 provides an overview of sessions three and four, which were the supplement treatment sessions.

### Upper body power

The medicine ball put is a commonly used field test used to measure upper body explosive power specific to functional movements such as basketball passes and the rapid punching of combat athletes. All medicine ball put tests during this investigation were conducted using the protocol set forth by Clemons and colleagues [20]. A 45° incline bench, 9 kg medicine ball, measuring tape, and chalk were used to administer and record test results. Following a specified upper body warm-up, subjects sat comfortably on the incline bench with feet flat on the floor and medicine ball grasped on each side and placed against the chest. The subject then attempted to propel the medicine ball at a 45° trajectory for maximal distance. Each subject was permitted three medicine ball put attempts with a 2 min rest in-between each attempt. A study staff member recorded each attempt to the nearest centimeter by measuring the closest chalk mark in the direction of the bench.

### Lower body power

The vertical jump assessment is a lower body explosive power test that compares favorably to isokinetic testing as a measurement of knee extension power [21]. A jump and reach test device (Vertec, Sports Imports, Hilliard, OH) was used to measure how high each subject jumped and reached to touch an overhead swivel vane. Subjects began in a standing position with an upright posture and feet shoulder width apart, then progressed into a semi-squat position while concurrently swinging arms backwards in preparation for the jump. The subject’s arms then swung forward in a simultaneous motion above the head as the individual jumped straight up in the air, reached to displace the vanes with an overhead swinging motion, and then landed. The highest displaced vane determined the maximum jump height. Each subject was permitted three vertical jump attempts with a 2 min rest in-between each attempt.

### Upper body strength

All 1-RM bench press strength testing was conducted using the protocol developed for this current study. Each subject performed a warm-up set using a standard barbell for a total of 12 repetitions. Next, subjects performed another warm-up set with a load that was approximately 75 % of their perceived maximum, and then completed 3–4 subsequent trials of increasing intensity to determine their 1-RM bench press. A rest period of 2 min was provided between each set. Testing occurred in the standard supine position with 5 points of contact on the bench. The subject lowered an Olympic barbell under control to mid-chest level and then pressed the weighted barbell until elbows were fully extended for a successful lift.

### Anaerobic power

To measure anaerobic power performance, subjects performed a Wingate anaerobic cycle test (Monark 894E, Vansbro, Sweden). Subjects performed a warm-up, which consisted of pedaling at approximately 60 rpm for 2 min interspersed with an all-out 2-second sprint at the end of the first minute. At the end of the second minute, subjects performed another all-out sprint lasting 30 s in duration against a constant force relative to individual body weight (7.5 % of body weight in kilograms). Subjects were instructed to remain seated throughout the entire 30-second sprint. Upon completion of the WAnT, subjects were instructed to perform a 2-minute cool down. Values for peak power, mean power, minimum power and fatigue index were measured and recorded during the 30 s testing period. Peak power was defined as the highest mechanical power output recorded during the test. Mean power was defined as the average mechanical power output recorded during the test. Minimum power was defined as the lowest mechanical power output recorded during the test. Fatigue index was calculated by dividing the difference between the highest mechanical power output and the lowest mechanical power output by the highest mechanical power output, and then multiplying by 100 to determine a percentage.

### Supplement

**Fig. 2:**
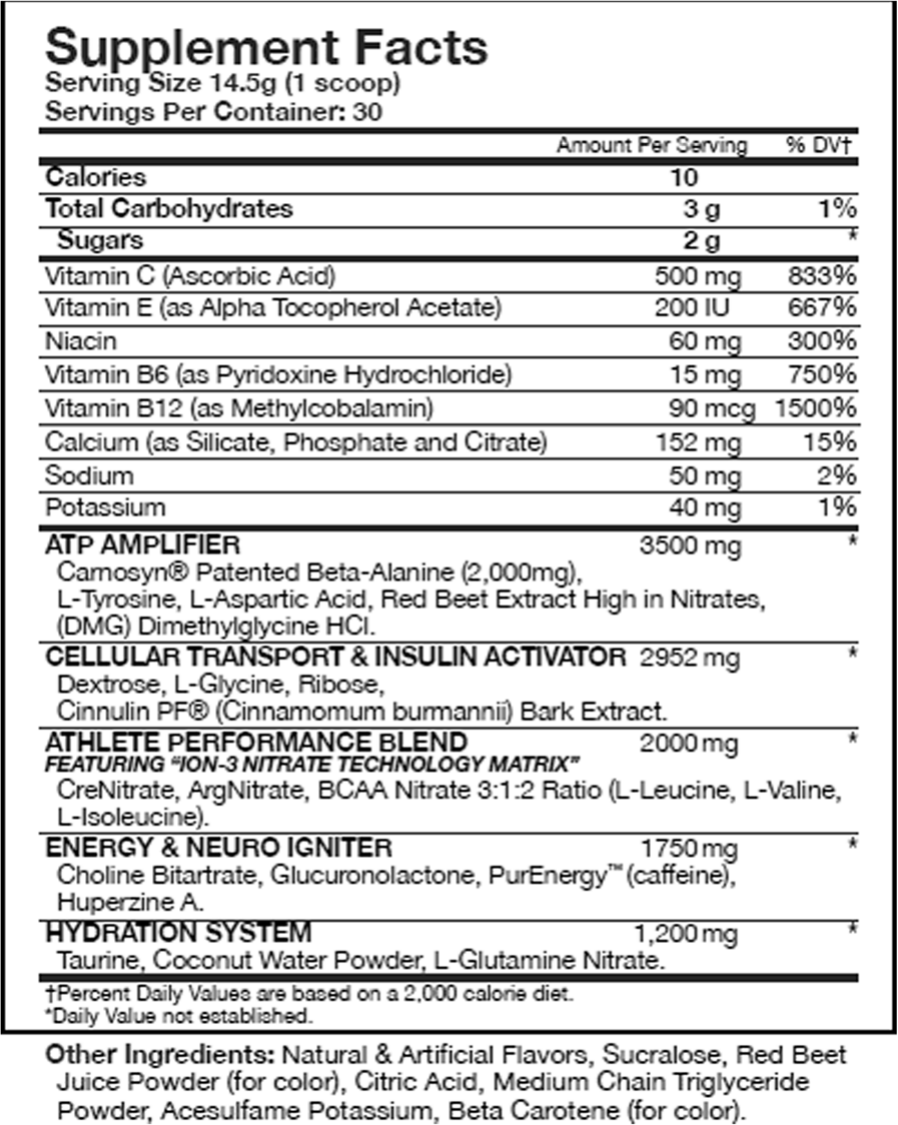
Pre-workout dietary supplement ingredients

During the third and fourth trials, subjects ingested either the supplement or a placebo powder mixed with 10 ounces of water. The pre-workout supplement, commercially available as Assault™, consisted of 1 scoop or 14.5 g of powder containing 10 calories, 3 g of carbohydrate, and 0 g of fat. Figure 2 lists the ingredients contained in this pre-workout dietary supplement. The placebo consisted of flavored maltodextrin, which was designed to appear and taste similar to the actual supplement. Subjects were permitted cold water *ad libitum* throughout each trial.

### Statistical analysis

Data analyses were performed using the computer software program SPSS version 22.0. Descriptive characteristics of the sample were analyzed. All performance data were analyzed using a 1-factor [1x3] within-subjects repeated measures analysis of variance (RM ANOVA). Post-hoc tests were analyzed via paired samples t-tests. The alpha criterion was set at a p-value less than 0.05. Mean differences were used to calculate Cohen’s d as an effect size. All assumptions were met and the data normally distributed.

## Results

**Fig. 3:**
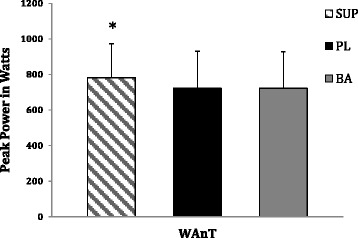
WAnT anaerobic peak power in watts. Note. Data are presented as mean ± standard deviations. Notation indicates statistically significant differences at (*p* < 0.05). * = significantly greater than baseline and placebo

**Fig. 4:**
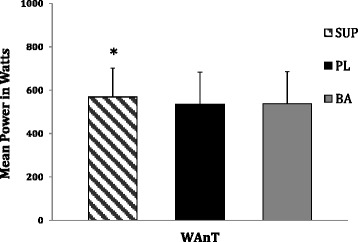
WAnT anaerobic mean power in watts. Note. Data are presented as mean ± standard deviations. Notation indicates statistically significant differences at (*p* < 0.05). * = significantly greater than baseline and placebo

The RM ANOVA revealed a significant within-subjects effect for anaerobic peak power (*p* = 0.001), and anaerobic mean power (*p* = 0.007) relative to the WAnT. Post-hoc analyses revealed that the SUP provided a significant increase in anaerobic peak power (782 ± 191 W) and mean power (569 ± 133 W) in comparison to the PL (Peak Power = 722 ± 208 W; *p* = 0.003; effect size = 0.30), (Mean Power = 535 ± 149 W; *p* = 0.006; effect size = 0.24), and baseline trials (Peak Power = 723 ± 205 W; *p* = 0.01; effect size = 0.28), (Mean Power = 535 ± 148 W; *p* = 0.02; effect size = 0.22), respectively (refer to Figs. 3 and 4). Table 1 demonstrates the raw data (mean ± SD) in watts for anaerobic peak, mean, and minimum power, as well as fatigue index relative to the WAnT for each treatment group. No significant within-subjects effects were observed for the medicine ball put (*p* = 0.31), vertical jump (*p* = 0.15), 1-RM bench press (*p* = 0.42) or fatigue index (*p* = 0.85).

**Table 1:** Measures of Wingate anaerobic capacity performance and fatigue index

	Baseline	Placebo	PWS	Effect size		Difference
				PWS-Baseline	PWS-Placebo	
Peak Power (W)	723 ± 205	722 ± 208	782 ± 191*	0.28	0.3	Small
Mean Power (W)	538 ± 148	535 ± 149	569 ± 133*	0.22	0.24	Small
Minimum Power (W)	331 ± 90	336 ± 87	356 ± 83	0.29	0.24	Small
Fatigue Index (%)	52.8 ± 6.6	53.3 ± 8.4	53.6 ± 8.7	0.10	0.03	Trivial

*Note*: Data are presented as mean ± standard deviation. *PWS* pre-workout supplement. * = significantly greater than baseline and placebo (*p* < 0.05). Effect sizes, < 0.2 = trivial; 0.2 to 0.49 = small; 0.5 to 0.79 = moderate; > 0.8 = large

## Discussion

This investigation examined the effects of a commercially available pre-workout supplement on measures of anaerobic power, upper and lower body explosive power, and upper body strength in a recreationally active population. The results of this study indicate that consumption of this pre-workout dietary supplement can significantly improve both anaerobic peak power and anaerobic mean power in comparison to a placebo treatment. Supplement ingestion did not reveal any significant ergogenic benefit for upper and lower body explosive power or upper body strength. Recent scientific investigations may provide further insight into caffeine’s effects on explosive power and maximal strength. Tucker and colleagues [22] found that ingesting 3 mg · kg^−1^ of caffeine did not significantly improve the lower body power of basketball athletes during vertical jump testing, which is similar to the findings in this study. Another study found that a relatively high dose of caffeine (9 mg · kg^−1^) can produce an ergogenic effect on 1-RM bench press performance, but may also result in adverse side effects [23]. Therefore, it is possible that the recommended caffeine dosage provided to subjects in this study may not have been large enough to facilitate a potential stimulatory effect on explosive power or upper body strength.

The active ingredients that comprise the ‘ATP Amplifier Blend’ (refer to Fig. 2) in the pre-workout dietary supplement include beta-alanine, L-tyrosine, L-aspartic acid, red beet extract, and dimethylglycine HCL. Beta-alanine supplementation increases the concentration of muscle carnosine, which acts as an intracellular buffer allowing for improved performance during high intensity exercise lasting 1–4 minutes, when acidosis is highest [24]. While beet extract and dimethylglycine HCL are both theorized to improve mitochondrial efficiency, recent research demonstrated no significant differences between supplement and placebo groups when measuring the effect of beet extract [25] and dimethylglycine HCL on anaerobic power [26]. Tyrosine is a nonessential amino acid that is essential for the production of catecholamine neurotransmitters, including dopamine, epinephrine, and norepinephrine. While recent research has reported that pre-exercise tyrosine supplementation improves cognitive function during soccer specific exercise in a warm environment, there is little evidence for tyrosine improving anaerobic power or acting more globally as an ergogenic aid [1, 27].

The active ingredients that comprise the ‘Athlete Performance Blend’ (refer to Fig. 2) include creatine, arginine-nitrate, and BCAAs. Arginine-nitrate is thought to mediate a tolerance build-up for nitrate, thus allowing for continual vasodilation of the blood vessels. Research conducted by Olek and colleagues [28] demonstrated that 2 g of arginine did not improve anaerobic power as measured during the Wingate Anaerobic Cycle Test. While the evidence is limited regarding the influence of BCAAs on anaerobic performance, Fukuda et al., [29] demonstrated that a combination of caffeine, creatine, and BCAAs did have an acute effect on anaerobic performance, which supports previous findings relative to caffeine supplementation and anaerobic power performance [12]. Both beta-alanine and creatine are active ingredients in the pre-workout dietary supplement used in this study, which have been shown to independently improve high intensity performance following long-term usage. While creatine functions primarily by increasing creatine phosphate stores to be used for energy during high intensity activities, beta-alanine is thought to increase buffering capacity by increasing carnosine concentrations, thus inhibiting the accumulation of H^+^ ions. However, manufacturers of both creatine and beta-alanine often recommend several weeks of loading to enhance exercise performance. Notably, this study did not include a loading phase, and utilized a design that measured only acute ingestion of the caffeine-containing pre-workout dietary supplement.

The active ingredients that comprise the ‘Energy and Neuro Igniter Blend’ (refer to Fig. 2) include choline-bitartrate, glucuronolactone, caffeine, and huperzine A. Choline-bitartrate is a chemical compound, which is thought to augment acetylcholine neurotransmission, thus enhancing muscle fiber recruitment. Huperzine A is a chemical compound that has been shown to inhibit activity of acetylcholinesterase, an enzyme that breaks down acetylcholine [30]. Therefore, some could theorize that the combination of choline-bitartrate and huperzine A may optimize acetylcholine release and improve muscle fiber recruitment. While evidence suggests that choline depletion during exercise may limit performance due to the inability to delay fatigue, research conducted by Spector et al., [31] and Warber et al., [32] concluded that choline levels do not drop during exercise and that choline supplementation is ineffective at delaying fatigue. Glucuronolactone is a natural compound, which is believed to increase endurance capacity [33, 34]. While evidence is lacking relative to its impact on anaerobic exercise performance, Forbes et al., [35] reported that a commercially available energy drink containing 15 mg of glucuronolactone did not have a significant impact on anaerobic power.

Caffeine is a mild nervous system stimulant with effects similar to amphetamines, only much weaker [36]. Caffeine, the principal active ingredient in the pre-workout dietary supplement used in this investigation has been shown to enhance aerobic endurance performance through augmenting fat oxidation, thus preserving muscle glycogen content and increasing time to exhaustion [11]. However, the relationship between caffeine and anaerobic performance remains equivocal. Reports of increases in anaerobic peak power following caffeine supplementation are believed to occur through increased motor unit firing rates, mobilization of calcium ions from the sarcoplasmic reticulum, and surges in nitric oxide concentrations [12]. In contrast, other studies have reported no differences when examining the effects of caffeine on anaerobic power [15, 16, 37]. Since the pre-workout dietary supplement used in this investigation is a proprietary blend (i.e., specific dosages are not listed), it is difficult to determine whether the principle ingredient caffeine, or any other individual ingredient was primarily responsible for the increases in anaerobic peak and mean power demonstrated in the current study. While acute ingestion of caffeine has been shown to favorably impact high intensity exercise, many of the other ingredients contained in this study’s pre-workout supplement have been shown to be ineffective at improving anaerobic performance independently. Therefore, it can be postulated that caffeine, the principle active ingredient in the pre-workout supplement used in this study, likely facilitated an ergogenic effect for anaerobic power performance, and when combined with various other ingredients may have had a synergistic effect, thus enhancing the ergogenic benefit and stimulatory potential.

## Conclusions

Considerable attention has been paid to the use of pre-workout supplements purported to enhance exercise performance. The results of this study indicate that acute ingestion of the commercially available pre-workout dietary supplement can significantly improve both anaerobic peak power and mean power in recreationally trained males. The ingestion of the pre-workout dietary supplement and the resulting significant increases in anaerobic power observed in this study came with no adverse side effects. In contrast, the pre-workout supplement did not improve upper body power, lower body power, or maximal bench press strength. Given the scarcity of research on pre-workout supplements, more research is warranted to gain a better understanding of their effects on anaerobic modes of exercise.

## Abbreviations

1-RM, One-rep maximum bench press; BA, Baseline; BCAAs, Branched-chain amino acids; MBP, Medicine ball put; PL, Placebo; RM ANOVA, Repeated measures analysis of variance; SUP, Pre-workout supplement; VJ, Vertical jump test; WAnT, Wingate anaerobic power test
